# Continuing Professional Development – Medical Imaging

**DOI:** 10.1002/jmrs.719

**Published:** 2023-08-21

Maximise your CPD by reading the following selected article and answer the five questions. Please remember to self‐claim your CPD and retain your supporting evidence. Answers will be available via the QR code and online at www.asmirt.org/news‐and‐publications/jmrs, as well as published in JMRS – Volume 71, Issue 4 December 2024.

## Medical Imaging—Original Article

### The cost of perfection: An investigation into the unnecessary rejection of clinically acceptable lateral wrist imaging

Steward A, Semsem S, Currie K, Bentley L, Mineo R, McAnulty K, Master V, Holliday M. (2023) *J Med Radiat Sci*. https://doi.org/10.1002/jmrs.702

1. In this study, the department reject rate for lateral wrist imaging was found to be 38.7%. This is how many times greater than that accepted in the literature?
  - 2 times.
  - 3 times.
  - 5 times.
  - The same.
2. In this study, what was the unnecessary repeat rate for lateral wrist imaging?
  - 40%–50%
  - 50%–60%
  - 70%–80%
  - 80%–90%
3. Junior radiographers were how much more likely to seek to repeat imaging than radiologists?
  - >30%
  - >50%
  - >60%
  - >70%
4. What outcome lead to the consideration that the inflated reject rate was the result of a lack of clinical understanding by junior radiographers rather than the ability to correctly position patients?
  - When the unnecessary repeat rate was taken from the actual reject rate, it fit within the accepted parameters described in literature.
  - Junior radiographers performed comparably to the experienced radiographers.
  - Junior radiographers were unable to describe their reasoning for wanting repeat imaging.
  - When reviewing the rejected imaging, all junior radiographers agreed that repeat imaging was warranted for only 11 of the 162 images.
5. Among the reasons for the requirement of repeat imaging suggested by radiologists, one was not considered by any of the radiographers. Which reason was this?
  - Over exposure of the image.
  - Artefacts obscuring the image.
  - Adequate projection through the joint space.
  - Overcollimation that did not include required anatomy.

**Recommended further reading:**

1. Decoster R, Toomey R, Smits D, Haygood TM, Ryan ML. Understanding reasons for image rejection by radiologists and radiographers. *J Med Radiat Sci*. 2023 Jun;70(2):127–136. doi: 10.1002/jmrs.641.
2. Atkinson S, Neep M, Starkey D. Reject rate analysis in digital radiography: an Australian emergency imaging department case study. *J Med Radiat Sci*. 2020 Mar;67(1):72–79. doi: 10.1002/jmrs.343.
3. Neep MJ. Making the most of X‐ray reject analysis in the digital age. *J Med Radiat Sci*. 2023 Jun;70(2):109–111. doi: 10.1002/jmrs.680.

## Answers


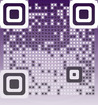


Scan this QR code to find the answers, or visit www.asmirt.org/news‐and‐publications/jmrs

